# YAP/TAZ deletion in vascular smooth muscle cells mirrors atherosclerosis-associated transcriptional programs^☆^

**DOI:** 10.1016/j.jmccpl.2025.100487

**Published:** 2025-10-05

**Authors:** Fatima Daoud, Johan Holmberg, Hanna Winter, Nadja Sachs, Lars Maegdefessel, Sebastian Albinsson

**Affiliations:** a Department of Experimental Medical Science, Lund University, 22184, Lund, Sweden; b Department of Physiology and Biochemistry, University of Jordan, 11942 Amman, Jordan; c Institute of Molecular Vascular Medicine, Technical University of Munich, Munich, Germany; d Department for Vascular and Endovascular Surgery, Technical University of Munich, 80333, Munich, Germany; e German Center for Cardiovascular Research DZHK, Partner Site Munich Heart Alliance, 80336, Berlin, Germany; f Department of Medicine, Cardiovascular Unit, Karolinska Institute, 17177, Stockholm, Sweden

**Keywords:** Hippo signaling, Transdifferentiation, Chondrogenesis, Vascular remodeling, Vascular calcification, Serum response factor

## Abstract

The transcriptional co-activators YAP (*YAP1*) and TAZ (*WWTR1*) are central regulators of vascular smooth muscle cell (VSMC) phenotype and vascular homeostasis. This study investigates the consequences of VSMC-specific YAP/TAZ deletion and its relevance to atherosclerosis. Using bulk and single-cell RNA sequencing data, we demonstrate that gene expression changes following two (2-week YT) and eight weeks (8-week YT) of YAP/TAZ deletion recapitulate key features of murine and human atherosclerosis. Transcriptomic comparisons revealed substantial overlap and concordance between YAP/TAZ-deficient VSMCs and different stages of plaque development, with 8-week YT displaying stronger resemblance to atherosclerotic lesions. Shared differentially expressed genes were enriched for inflammatory mediators, extracellular matrix remodeling factors, and chondrogenic markers. Gene ontology and Reactome pathway enrichment analyses highlighted upregulation of immune-related pathways, extracellular matrix remodeling, and chondrogenic differentiation, accompanied by the downregulation of muscle contractile programs. Integration of ChIP-seq data and promoter motif analyses identified 19 conserved YAP–TEAD target genes that were consistently repressed at both 2-week and 8-week YT. Several of these target genes were also downregulated in atherosclerotic plaques, such as genes involved in cytoskeletal integrity (e.g., *SRF, NEXN*). Notably, loss of YAP/TAZ induced a phenotypic shift in VSMCs toward chondromyocyte-like and fibromyocyte-like states, analogous to those seen in murine and human atherosclerosis. These findings suggest that YAP/TAZ safeguard VSMC identity by directly repressing pro-inflammatory and osteochondrogenic programs, and that their disruption may contribute to atherogenesis. This positions YAP/TAZ–TEAD axis as a key guardian of vascular homeostasis and a potential therapeutic target for limiting plaque progression.

## Graphical abstract

**Figure:**
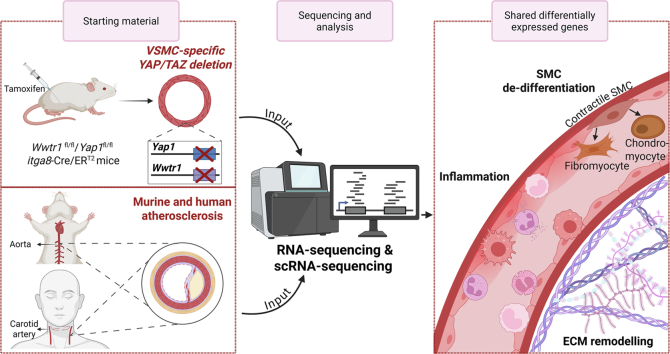

## 1 Introduction

Atherosclerosis is a chronic multifactorial process driven by the interplay of genetic predisposition and environmental risk factors. Hypercholesterolemia, hypertension, diabetes, and smoking are major contributors triggering endothelial dysfunction, lipid accumulation, and inflammation. This leads to the formation of fatty streaks that gradually progress into fibrous plaques. Atherosclerosis may remain silent until the plaque stenosis exceeds 70 % of blood vessel diameter which reduces blood flow to the designated organ [1]. Moreover, rupture or erosion of the plaque can lead to thrombosis and sudden events like myocardial infarction or stroke. Therefore, cardiovascular diseases remain the leading cause of mortality worldwide, accounting for roughly one-third of all deaths [2].

In addition to lipid accumulation and immune cell infiltration, there is growing recognition of the crucial role played by vascular smooth muscle cells (VSMCs) in atherogenesis and plaque progression [3]. Although VSMCs were traditionally viewed as stabilizing components of atherosclerotic plaques through their role in forming the fibrous cap, recent advances including lineage tracing and single-cell RNA sequencing (scRNA-seq) have demonstrated that VSMC-derived cells play a substantial role in increasing plaque complexity [4]. During atherogenesis, VSMCs undergo phenotypic switching, a process wherein mature contractile smooth muscle cells (SMC) de-differentiate and assume alternative cell fates such as macrophage-like or osteochondrogenic phenotypes. The latter is characterized by down-regulation of contractile SMC markers, expression of bone- or cartilage-associated genes, and deposition of mineralized matrix. In murine models, smooth muscle-derived cells account for up to 70 % of foam cells, indicating a highly plastic and multifaceted role for VSMCs in plaque pathogenesis [5,6]. Similar cell heterogeneity was found in atherosclerotic plaques from human carotid and coronary arteries [7].

The osteochondrogenic transdifferentiation of VSMCs is recognized as a pivotal process in atherosclerotic calcification [8]. Several signaling pathways have been recognized to be involved in this transdifferentiation such as, Wnt/β-catenin pathway, BMP/SMAD pathway, and NF-kβ signaling pathway. Besides these, the Hippo pathway and its downstream effectors Yes-associated protein (YAP) and transcriptional co-activator TAZ have drawn considerable attention recently for their role in maintaining VSMC contractile phenotype and suppression of osteochondrogenic transdifferentiation [9,10]. YAP/TAZ are transcriptional co-activators that respond to mechanical cues, such as cyclic stretch and matrix stiffness, and metabolic factors by translocating to the nucleus and modulating gene expression [11,12]. Using inducible and SMC-specific deletion of YAP/TAZ, we have established their crucial role in vascular function and protection against vascular diseases [13]. Strikingly, two weeks of VSMC-specific YAP/TAZ deletion (2-week YT) is sufficient to develop aortic aneurysm characterized by chondrogenic transdifferentiation and vascular inflammation [10]. These findings implicate YAP/TAZ as critical guardians of smooth muscle contractile differentiation and vascular health.

Emerging evidence from both animal models and human studies suggests that YAP/TAZ-regulated genes serve as important modulators of VSMC behavior [9,10]. Notably, the gene signature of YAP/TAZ-dependent transcription in VSMCs is beginning to be characterized and may vary between cell types. In this study, we aim to investigate how the YAP/TAZ regulon contributes to gene expression changes in atherosclerosis using mouse and human RNA sequencing data (RNA-seq), which will provide valuable insights into the mechanisms of atherogenesis and may unveil novel targets that can be used to prevent and treat atherosclerosis.

## 2 Methods

### 2.1 Used datasets in this paper (Table 1)

#### 2.1.1 Bulk RNA-seq data of VSMC-specific YAP/TAZ deletion for 2 and 8 weeks (GSE240233)

These datasets are published with our seminal work on YAP/TAZ (GSE240233) [10]. Briefly, *Itga8*-CreER^T2^ mice were bred into mice with flanked loxP for both *Yap1* (YAP) and *Wwtr1* (TAZ). At age of 8 weeks, these mice were given tamoxifen to induce Cre recombination which resulted in VSMC-specific YAP/TAZ deletion. Mice were sacrificed after two (2-week YT) or eight weeks (8-week YT) of the first tamoxifen injection and RNA-seq was performed on thoracic aortae from the knockout and control mice.

**Table 1:** Summary of the used datasets. This table lists the publicly available and in-house datasets analyzed in this study. It includes details on sequencing platform, species, vascular tissue analyzed, experimental model or procedure, and the specific subgroups used in the comparisons.

Dataset Accession	Sequencing Method	Species	Tissue	Experimental Procedure / Model	Subgroups / Comparisons
GSE240233	Bulk RNA-seq	Mouse	Thoracic aorta	VSMC-specific inducible YAP/TAZ knockout: Itga8-CreER^T2^ mice crossed with *Yap1*^^fl/fl^;*Wwtr1*^^fl/fl^, treated with tamoxifen. Control: Age-matched Cre-negative floxed mice.	2-week YT, 8-week YT
GSE205929	Bulk RNA-seq	Mouse	Aorta	Atherosclerosis model: LDL receptor-deficient mice, expressing only apolipoprotein B100, fed high-fat diet (HFD). Control: Age-matched wild-type C57BL/6J mice on a standard chow diet.	Early AS (1 month HFD), Late AS (3 months HFD)
GSE131776	scRNA-seq	Mouse	Aortic root & ascending aorta	ApoE^−/−^;*Myh11*-CreER^T2^;ROSA-tdTomato mice fed HFD. SMC-derived cells were sorted for single-cell RNA-seq after 8 and 16 weeks HFD	DEGs from 8- and 16-week HFD combined
E-MTAB-12019	scRNA-seq	Mouse	Aortic arches	*Myh11*-CreER^T2^ ROSA tdTomato mice. 4 weeks after tamoxifen injection, hypercholesterolemia was induced by PCSK9D377Y virus. Mice were then fed HFD for 16 weeks, followed by 12 weeks of low-fat diet plus weekly ApoB antisense oligonucleotides (ASO).	Atherosclerosis: 16 wK HFD. Treatment: 12 wK of LFD plus ApoB ASO
GSE28829	Bulk RNA-seq, Human_AS_1	Human	Carotid atherosclerotic plaques	Early-stage plaques (intimal thickening/xanthoma, n = 13). Advanced plaques (fibrous cap, n = 16)	Advanced AS vs. early
Expanded datasets of PMID: 37797407	Bulk RNA-seq, Human_AS_2	Human	Carotid atherosclerotic plaques	Lesions were histopathologically classified as early (n = 54) or advanced (n = 200)	Advanced AS vs. early

#### 2.1.2 Bulk RNA-seq data of early and late murine atherosclerosis (GSE205929)

Örd et al. published bulk RNA-seq data from mouse aorta (Gene Expression Omnibus accession number: GSE205929). In their study, mice deficient in the low-density lipoprotein receptor and expressing only apolipoprotein B100 were fed a high-fat diet for either 1 or 3 months to model early and advanced stages of atherosclerosis, respectively. Age-matched wild-type C57BL/6 J mice on a standard chow diet were used as controls [14].

#### 2.1.3 scRNA-seq of murine atherosclerosis (GSE131776) (E-MTAB-12019)

scRNAseq was performed on cells isolated from the aortic root and ascending aorta of ApoE deficient mice fed a high fat diet. The mice had *Myh11*-CreER^T2^ and ROSA tdTomato alleles that allowed SMC lineage tracing [15] (GSE131776). Du et al. reanalyzed this dataset and compared gene expression of modulated SMC in comparison to contractile SMC and found 765 and 976 differentially expressed genes (DEGs, Padj <0.05) after 8 and 16 weeks of high fat diet, respectively [16]. Combining these gene lists resulted in 952 DEG thatwe have used in our comparison.

Further characterization and sub-clustering of SMC- related phenotypes in atherosclerosis using scRNA-seq were done by Carramolino et al. [17]. In their paper, they used linage tracing by using *Myh11*-CreER^T2^ ROSA tdTomato mice. Four weeks after tamoxifen injection, hypercholesterolemia was induced by injecting mice with PCSK9/D377Y virus particles. The mice then were put on a high fat diet for 16 weeks, after that one group of interest was converted to low fat diet plus weekly injections of antisense oligonucleotides targeting ApoB mRNA for 12 weeks. Datasets were deposited in BioStudies (ArrayExpress) with accession number (E-MTAB-12019).

#### 2.1.4 Advanced human atherosclerosis datasets (Human_AS_1: GSE28829) (Human_AS_2)

We reanalyzed a publicly available microarray dataset from the Gene Expression Omnibus (accession number GSE28829), which includes gene expression profiles from early (*n* = 13) and advanced (*n* = 16) human carotid atherosclerotic plaques. The tissue samples were obtained from the Maastricht Pathology Tissue Collection and categorized based on histopathological criteria. Lesions characterized by intimal thickening and intimal xanthoma were classified as early-stage plaques, whereas those exhibiting a fibrous cap atheroma were considered advanced stage.

We also utilized RNA-seq data (Human_AS_2) obtained from human carotid artery samples representing early (*n* = 54) and advanced (*n* = 200) atherosclerotic lesions. These samples were collected from patients undergoing carotid endarterectomy and provided by the Munich Vascular Biobank, as previously described [18,19].

### 2.2 Bioinformatics analyses

**Fig. 1:**
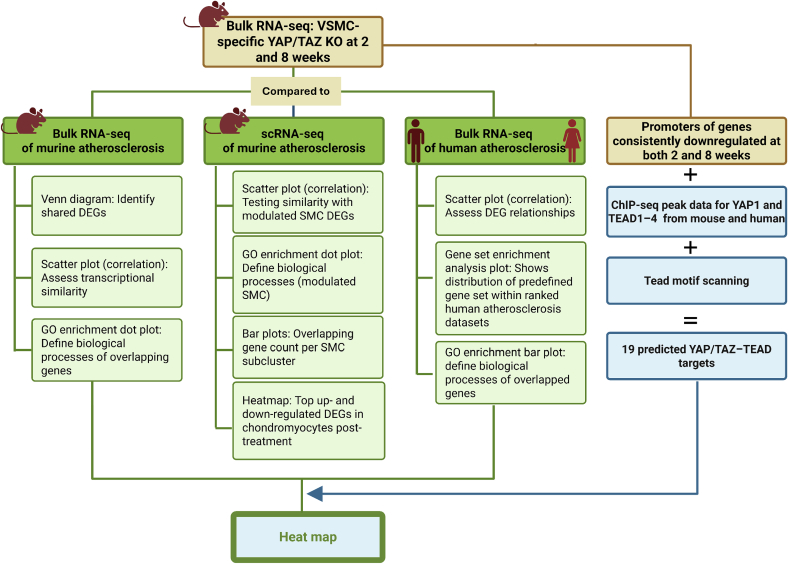
**Flow chart of the bioinformatics pipeline illustrating the comparisons made between different datasets, the relevant graphs, and goals.** DEG, differentially expressed genes with Padj < 0.05; GO, Gene Ontology; SMC, smooth muscle cell.

An overview of the bioinformatics workflow, including the specific goals of each dataset comparison, is summarized in (Fig. 1).

#### 2.2.1 YT gene set overlap with GSE205929

DEGs were defined as those with Padj <0.05, and the background list was defined as the union of all analyzed genes. To assess the overlap between DEGs from YAP/TAZ-deficient mice and atherosclerotic samples, we performed one-sided hypergeometric tests in R, based on observed and expected overlaps. Multiple testing correction was applied using the Benjamini-Hochberg method. Venn diagrams were generated using the VennDiagram package to visualize intersections between DEG sets.

#### 2.2.2 Correlation analysis between YT DEGs and GSE205929

To assess transcriptional similarity between YAP/TAZ-deficient VSMCs and murine atherosclerosis, we computed pairwise Spearman correlations of log_2_fold changes for overlapping DEGs between each YAP/TAZ deletion point (2 or 8 weeks) and early or late atherosclerosis. The top 10 upregulated genes in the YAP/TAZ dataset were highlighted in scatterplots along with linear regression curve.

#### 2.2.3 Gene ontology (GO) and reactome pathway enrichment analyses on YT DEGs and GSE205929

We performed GO enrichment on genes upregulated in both the YAP/TAZ deletion and atherosclerosis datasets. Upregulated genes were defined as those with positive log_2_fold change in both datasets and Padj <0.05. GO biological process enrichment was performed using enrichGO from the clusterProfiler R package, applying Benjamini-Hochberg correction with a significance threshold of Padj <0.05. Redundant terms were simplified with 0.7 as cutoff. Enriched terms were visualized ggplot2-based dot plots to compare GO term enrichment between 2-week and 8-week YT for both early and late atherosclerosis overlaps. Reactome pathway enrichment analyses were performed on DEGs shared between 2- and 8-week YAP/TAZ deletion and either early or late atherosclerosis. The analyses were conducted using the Reactome Pathway Browser available at https://reactome.org.

#### 2.2.4 Correlation, GO enrichment, and reactome pathway analyses between 8-week YT and modulated SMC (GSE131776)

Transcriptomic alignment between 8-week YT and the modulated SMC phenotype was evaluated by comparing DEGs with Padj <0.05 from both datasets. The modulated SMC gene list was derived from scRNA-seq of aortic SMCs in ApoE-deficient mice (GSE131776), as published by Du et al. [16]. Log_2_fold change values were extracted for overlapping DEGs, and pairwise Spearman correlation was calculated. Scatterplots were generated with ggplot2, with a regression line and correlation statistics added. The top 10 upregulated genes in 8-week YT were highlighted in red. Overlapping DEGs were analyzed using GO enrichment for biological processes and Reactome pathway analysis, as previously described.

#### 2.2.5 Cluster enrichment analysis using SMC subtypes (E-MTAB-12019)

To assess enrichment of YAP/TAZ-regulated genes across SMC subtypes, we compared upregulated DEGs from 2-week and 8-week YT with cluster-specific markers (E-MTAB-12019). DEGs (Padj <0.05, log_2_FC > 0) were matched by gene symbols to cluster markers, and overlapping genes were identified for each SMC subtype. The number of overlapping genes per cluster was tested for enrichment using one-sided hypergeometric tests, using all expressed genes as the background. P values were corrected using Benjamini-Hochberg method.

#### 2.2.6 8-week YT comparison with chondromyocyte response to lipid-lowering therapy (E-MTAB-12019)

To examine whether YAP/TAZ-regulon in VSMCs aligned with transcriptional changes in chondromyocytes following cholesterol-lowering intervention, we compared DEGs from 8-week YT with cell type-specific DEGs identified after 12 weeks of lipid-lowering treatment (E-MTAB-12019) [17]. Overlapping DEGs within the chondromyocyte cluster were extracted and stratified by direction of regulation in 8-week YT (up or down). For each group, 25 most strongly downregulated or upregulated were visualized using pheatmap, with unified color scaling to enable comparison.

#### 2.2.7 Cross-species 8-week YT comparison with human atherosclerotic plaques (GSE28829)

To evaluate the conservation of YAP/TAZ-regulated transcriptional signatures in human atherosclerosis, we compared DEGs from 8-week YT RNA-seq with gene expression profiles from human carotid plaques (GSE28829). Gene symbols were harmonized to the uppercase, and DEGs with Padj <0.05 were retained. Spearman correlation was computed for overlapping DEGs and visualized using scatter plots with top DEGs in 8-week YT labeled.

To determine whether genes regulated by 8-week YT deletion were enriched among DEGs in human plaques, we conducted Fisher's exact tests and Gene Set Enrichment Analysis (GSEA) using the fgseaMultilevel method in R. Specifically, gene sets identified as up- or downregulated in the 8-week YT dataset were evaluated against a ranked list of human plaque genes sorted by log_2_fold change. Shared up- and downregulated genes between datasets were further investigated for functional enrichment of biological processes using GO and Reactome pathways.

#### 2.2.8 YAP/TAZ–TEAD target prediction pipeline

To identify putative direct transcriptional targets of YAP/TAZ in VSMCs, we integrated cross-species ChIP-seq data and promoter motif analysis. ChIP-seq peak data for YAP1 and TEAD1–4 (TEA domain transcription factor 1‐–4) was obtained from ChIP-Atlas (aggregated across all cell types). Peaks overlapping ENCODE blacklist regions were removed, and promoter-associated binding sites were defined as those located within ±2 kb of annotated transcription start sites using GenomicRanges. Each promoter-bound peak was scored using the narrowPeak score field (−log₁₀[q-value] × 10), reflecting binding strength and reproducibility. To categorize signal intensity, scores were classified using a five-color scale: blue: < 200, cyan: 200–499, green: 500–749, yellow: 750–999, and red: ≥ 1000. For each gene, the highest-scoring promoter peak was retained. Gene-level targets were mapped between human and mouse using BioMart ortholog annotations, yielding 1363 orthologous genes with promoter occupancy by both YAP1 and at least one TEAD factor in both species.

To refine this list, we performed promoter motif scanning using TEAD-specific position weight matrices from the JASPAR2022 database (TEAD1–4, vertebrates). Motif presence was assessed with motifmatchr, and genes with at least one TEAD-binding motif in promoter-bound peaks from either species were retained. This filtering step yielded 225 orthologous genes with both ChIP evidence and TEAD motif enrichment.

Finally, this list was intersected with genes consistently downregulated (Padj <0.05, log_2_FC < 0) at both 2- and 8-weeks following YAP/TAZ deletion in VSMCs. The resulting 19 genes represent a set of putative direct transcriptional targets of YAP/TAZ–TEAD signaling, supported by cross-species promoter binding, conserved TEAD motifs, and transcriptional repression in vivo*.*

#### 2.2.9 YAP/TAZ–TEAD panel in human and mouse atherosclerosis

To investigate whether the identified putative YAP/TAZ–TEAD target genes are modulated during atherosclerosis, we created a log_2_fold change matrix comprising 19 previously determined YAP/TAZ–TEAD targets. This matrix was generated by intersecting the target gene panel with existing atherosclerosis datasets, specifically GSE205929 (mouse early and advanced lesions), Human_AS_1: GSE28829, and Human_AS_2. A heatmap was subsequently produced to visualize gene expression patterns across disease stages and between species.

All analyses were performed in R version 4.4.2. Scripts and data are available upon request. A list of all gene abbreviations used in the article, together with their function, is provided in Supplementary Table 1.

## 3 Results

### 3.1 Shared gene signature in YAP/TAZ-deficient VSMCs and murine atherosclerosis

**Fig. 2:**
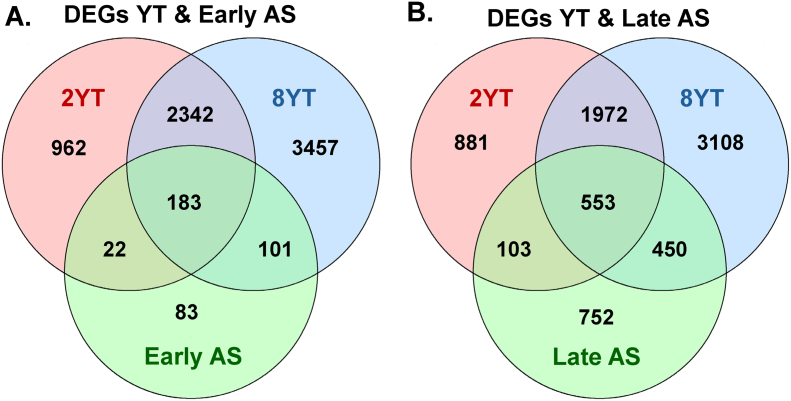
**Venn-diagram analysis of differentially expressed genes (DEG) in VSMCs-specific YAP/TAZ knockout mice at 2 and 8 weeks with early and late murine atherosclerosis.** **(A)** Overlap of DEGs identified in 2-week knockout mice (2YT; red), 8-week knockout mice (8YT; blue) and early murine atherosclerotic lesions (Early AS; green). **(B)** Overlap of DEGs identified in 2-week knockout mice (2YT; red), 8-week knockout mice (8YT; blue) and late murine atherosclerotic lesions (Late AS; green). DEG, differentially expressed genes with Padj < 0.05. (For interpretation of the references to color in this figure legend, the reader is referred to the web version of this article.)

To determine whether YAP/TAZ-regulated genes are differentially expressed in murine atherosclerosis, we compared RNA-seq data from 2-week and 8-week YT with bulk RNA-seq (GSE205929) of early and advanced atherosclerosis. DEGs from the 2-week YT samples overlapped with 205 genes associated with early atherosclerosis (52.7 %, *P* < 0.0001) (Fig. 2A). For the 8-week YT samples, the overlap increased to 284 genes (73 %, *P* < 0.0001) (Fig. 2A). When compared to the late atherosclerosis dataset, the overlap comprised 656 genes for 2-week YT (35.3 %, *P* < 0.0001) and 1003 genes for 8-week YT (54 %, *P* < 0.0001) (Fig. 2B). These findings suggest that YAP/TAZ-regulated transcriptional programs in VSMCs show a significant and progressive overlap with gene expression changes observed in murine atherosclerosis. The overlap is more pronounced at 8 weeks after YAP/TAZ deletion, proposing that prolonged loss of YAP/TAZ increasingly mirrors the molecular signatures of both early and advanced atherosclerotic stages.

To assess transcriptional concordance between the YT model and atherosclerosis, we compared the direction of regulation of DEGs. A total of 96.1 % of DEGs in 2-week YT and 95.1 % in 8-week YT samples showed the same direction of regulation as those in early atherosclerosis. Similarly, 89.3 % and 82.1 % of DEGs in the 2- and 8-week YT samples, respectively, were concordant with late-stage atherosclerosis. These findings indicate that the transcriptional responses in the YT model closely mirror gene regulatory changes in atherosclerosis, with particularly strong concordance at the early disease stage.

**Fig. 3:**
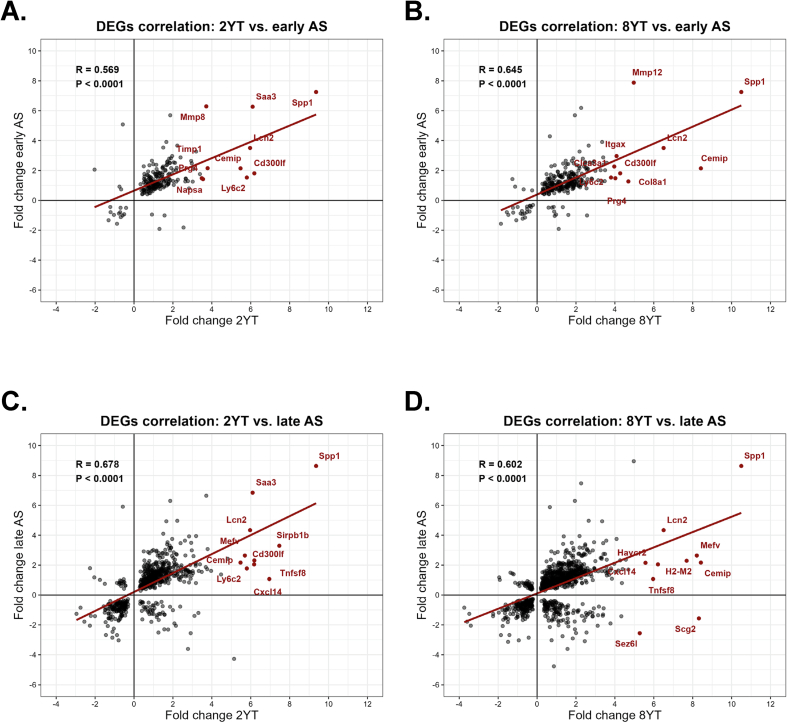
**Correlation of the shared differentially expressed genes (DEG) between VSMCs-specific YAP/TAZ knockout mice and murine atherosclerotic lesions at early and late stages.** Scatter plots compare log₂fold-changes of genes significantly dysregulated in YAP/TAZ-deficient VSMCs (YT) at two time points in YT (2 weeks [2YT], 8 weeks [8YT]) with those in murine atherosclerotic aortas (AS) at two stages (early, late). Panels: (**A)** 2YT vs. early AS. (**B)** 8YT vs. early AS. (**C)** 2YT vs. late AS. (**D)** 8YT vs. late AS. Each point represents a shared DEG, gray dots: all overlapping DEGs, red dots: the top 10 genes most strongly upregulated in YT. Solid red depicts the linear regression. R, Spearman coefficient; DEG, differentially expressed genes with Padj < 0.05. (For interpretation of the references to color in this figure legend, the reader is referred to the web version of this article.)

We assessed pairwise Spearman correlations between YAP/TAZ-deficient VSMC transcriptomes (2 and 8 weeks) and murine atherosclerosis (early, 1 month; late, 3 months) (Fig. 3A–D). Correlations were calculated on log_2_fold change of DEGs. All four comparisons (Fig. 3A:2wk–early, 3B: 8wk–early, 3C: 2wk–late, 3D: 8wk–late) showed positive, statistically significant associations (Spearman R ≈ 0.6). Among the upregulated genes in both datasets, *Spp1* (osteopontin) came on top of the list. *Spp1* is a pro-inflammatory cytokine that promotes vascular calcification and plays a role in VSMC phenotypic switching [[20], [21], [22], [23]]. Other top upregulated genes included *Saa3, Lcn2, Mefv,* and *Cxcl14*, all of which are potent inflammatory mediators, functioning as acute-phase proteins or chemokines [[24], [25], [26]]. Additionally, *Ly6c2, Sirpb1b, Cd300lf,* and *Tnfsf8* pointed toward immune cell infiltration. Alongside *Spp1, Cemip* is involved in extracellular matrix remodeling [27].

**Fig. 4:**
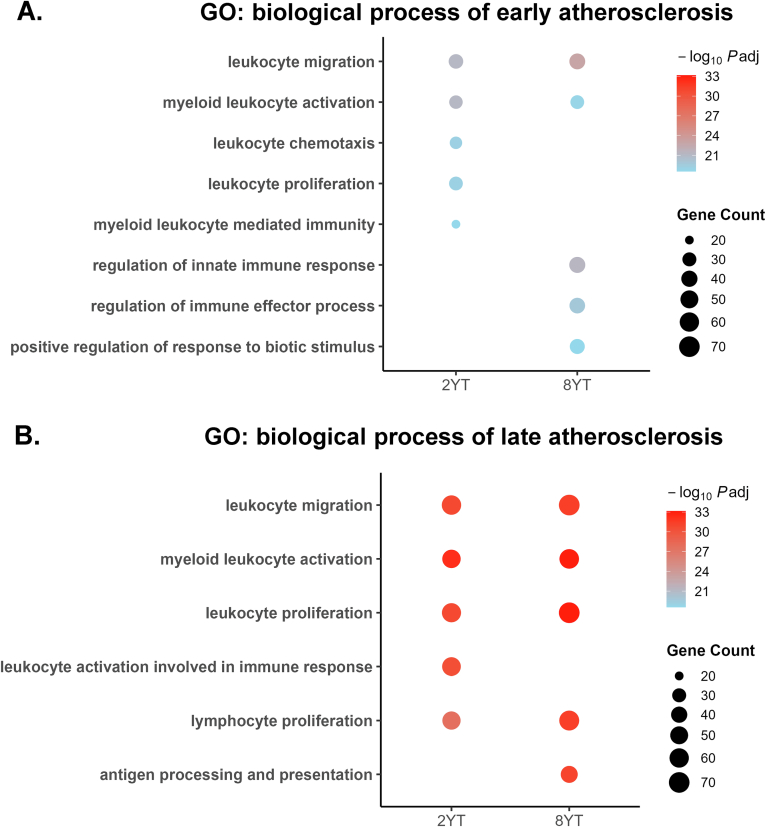
**Gene ontology (GO) of biological process enrichment in shared differentially expressed genes (DEGs) of early and late murine atherosclerotic and VSMCs-specific YAP/TAZ knockout mice at 2 and 8 weeks. A.** 2YT and 8YT with early atherosclerosis. **B.** 2YT and 8YT with late atherosclerosis. Each point represents one GO term; point size is proportional to the number of genes (“Gene Count”) annotated to that term, and point color reflects the statistical significance of enrichment (−log₁₀ adjusted P value). GO, Gene Ontology; Padj, Benjamini–Hochberg adjusted P value.

GO enrichment analysis indicates that deletion of YAP/TAZ in VSMC induces a pro-inflammatory and immune-activated transcriptional state, with substantial overlap with early atherosclerosis. As early as 2 weeks after YAP/TAZ deletion, there is significant enrichment of GO terms such as myeloid leukocyte activation, leukocyte migration, and leukocyte proliferation. The number of genes associated with these GO terms further increases at 8 weeks, suggesting a progressive amplification of the inflammatory response (Fig. 4A). Complementary Reactome pathway analysis of overlapping DEGs between YT and early atherosclerosis also revealed strong enrichment of immune-related pathways, including “Immune System”, “Innate Immune System”, “Cytokine Signaling”, and “Interleukin Signaling” (Table 2, Table S2).

**Table 2:** Reactome pathway analysis of overlapping genes between 2-week YT, 8-week YT, and early-stage murine atherosclerosis, showing the top 6 pathways.

Pathway identifier	Pathway name	Number of genes	FDR
R-HSA-168256	Immune System	107	6.92E-14
R-HSA-168249	Innate Immune System	56	1.41E-10
R-HSA-1280215	Cytokine Signaling in Immune system	51	5.41E-11
R-HSA-449147	Signaling by Interleukins	42	3.11E-13
R-HSA-1280218	Adaptive Immune System	32	4.09E-04
R-HSA-6798695	Neutrophil degranulation	31	8.86E-10

When comparing GO profiles from late-stage atherosclerosis with YT data, additional shared processes such as antigen processing and presentation and lymphocyte proliferation emerged. These findings suggest that adaptive immune mechanisms, including T-cell activation, become prominent features of both advanced atherosclerosis and the chronic YAP/TAZ-deficient state (Fig. 4B). Consistent with this, Reactome pathway analysis revealed similar enrichment of immune-related signaling pathways, such as cytokine and interleukin signaling, with a notably higher number of genes represented in each category (Table 3, Table S3). These results underscore a progressively intensified inflammatory and immunomodulatory environment associated with sustained YAP/TAZ deletion.

**Table 3:** Reactome pathway analysis of overlapping genes between 2-week YT, 8-week YT, and late-stage murine atherosclerosis, showing the top 6 pathways.

Pathway identifier	Pathway name	Number of genes	FDR
R-HSA-168256	Immune System	221	5.01E-14
R-HSA-168249	Innate Immune System	110	2.83E-09
R-HSA-1280215	Cytokine Signaling in Immune system	104	3.03E-12
R-HSA-449147	Signaling by Interleukins	83	5.01E-14
R-HSA-6798695	Neutrophil degranulation	63	1.46E-12
R-HSA-1280218	Adaptive Immune System	63	1.05E-02

### 3.2 YAP/TAZ deletion recapitulates atherosclerosis-associated VSMC phenotypes in scRNA-seq data

**Fig. 5:**
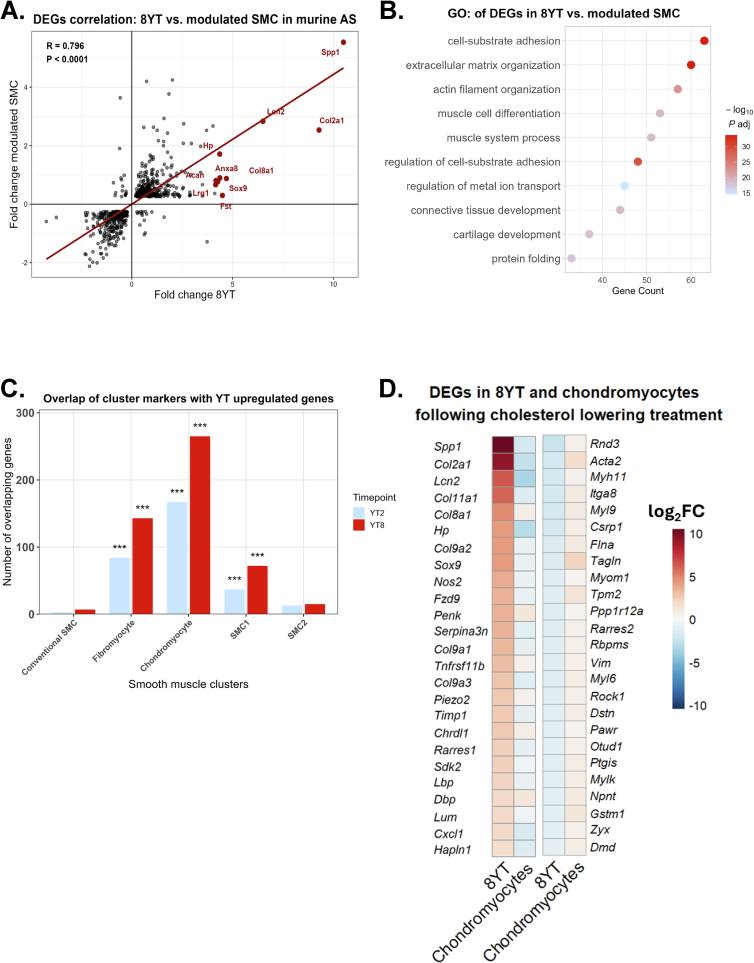
**Transcriptomic similarities between VSMCs YAP/TAZ-deficient and modulated VSMCs in atherosclerosis. (A)** Scatter plot comparing fold changes of differentially expressed genes (DEGs) between VSMCs-specific YAP/TAZ knockout mice at 8 weeks and modulated SMC from scRNA-seq data in murine atherosclerosis (GSE131776) [15]. The linear regression line and correlation coefficient are depicted. The most shared upregulated genes are highlighted in red. **(B)** GO enrichment analysis of overlapping DEGs from 8YT and modulated SMCs. Dot size indicates gene count; color scale represents –log₁₀Padj. **(C)** Bar graph showing the number of genes upregulated in VSMCs-specific YAP/TAZ knockout mice at 2 and 8 weeks that overlap with marker genes from five VSMC clusters identified by Carramolino et al. (E-MTAB-12019). Enrichment analysis was done using one-sided hypergeometric tests. P values were corrected using Benjamini-Hochberg method **(D)** Heatmap of top and bottom 25 DEGs in 8YT and chondromyocyte populations after cholesterol-lowering treatment. Color scale represents log₂fold change (log₂FC). GO, Gene Ontology; R, Spearman coefficient; DEG, differentially expressed genes with Padj < 0.05. (For interpretation of the references to color in this figure legend, the reader is referred to the web version of this article.)

To assess how YAP/TAZ-deficient VSMCs resemble transcriptional changes in atherosclerosis, we analyzed published scRNA-seq data from atherosclerosis in mouse models. Wirka et al. performed scRNA-seq on cells from the aortic root and ascending aorta of ApoE-deficient mice fed a high-fat diet (GSE131776). Smooth muscle cell lineage tracing was possible because of the presence of ROSA tdTomato alleles [15]. When compared to DEGs from 8-week YT model [10], we found a strong positive correlation between the overlapping genes (Fig. 5A). Notably, several genes involved in extracellular matrix organization and chondrogenic phenotype modulation, such as *Spp1, Col2a1, Lcn2, Sox9, Acan*, and *Col8a1*, were strongly upregulated in both datasets and are highlighted in red (Fig. 5A). These genes may represent shared markers of phenotypic switching toward a chondromyocyte-like state, further supporting the hypothesis that reduced YAP/TAZ activity in VSMCs promotes gene programs associated with vascular remodeling and atherosclerosis progression.

To elucidate the biological processes underlying the shared transcriptional signatures between YAP/TAZ-deficient VSMCs and modulated SMCs in atherosclerosis, we performed both GO and Reactome pathway enrichment analyses on the overlapping DEGs. These analyses consistently highlighted pathways involved in extracellular matrix remodeling, cytoskeletal reorganization, and phenotypic modulation of VSMCs. Specifically, GO enrichment revealed strong overrepresentation of terms such as cell-substrate adhesion, extracellular matrix organization, actin filament organization, muscle cell differentiation, and cartilage development, which are hallmarks of the transition from a contractile to a synthetic or chondromyocyte-like phenotype (Fig. 5B). Reactome pathway analysis further reinforced these findings, identifying significant enrichment of extracellular matrix organization, muscle contraction, degradation of the extracellular matrix and immune-related pathways including cytokine signaling and signaling by interleukins (Table 4, Table S4). Together, these results demonstrate that loss of YAP/TAZ triggers a transcriptional reprogramming that closely mirrors the pathological modulation of SMCs observed in atherosclerotic lesions.

**Table 4:** Reactome pathway analysis of overlapping genes between 8-week YT, and modulated SMC, showing the top 6 pathways.

Pathway identifier	Pathway name	Number of genes	FDR
R-HSA-1280215	Cytokine Signaling in Immune system	81	5.82E-03
R-HSA-1474244	Extracellular matrix organization	76	1.77E-13
R-HSA-449147	Signaling by Interleukins	51	2.04E-02
R-HSA-397014	Muscle contraction	38	3.12E-08
R-HSA-76002	Platelet activation, signaling and aggregation	38	6.65E-06
R-HSA-1474228	Degradation of the extracellular matrix	33	4.55E-10

To further refine the transcriptional profile resulting from YAP/TAZ deletion, we analyzed the dataset from Carramolino et al. (E-MTAB-12019) [17], which delineates five VSMC subclusters based on distinct marker gene expression: conventional SMCs (*Acta2, Myh11*), fibromyocytes (*Vcam1, Lum, Ly6a*), chondromyocytes (*Sox9, Col2a1*), SMC1 (characterized by intermediate levels of contractile markers and the pericyte marker *Rgs5*), and SMC2 (marked by high expression of contractile genes together with stress-response genes such as *Atf3* and *Fos*) [17]. In our analysis, genes upregulated in YAP/TAZ-deficient VSMCs were significantly enriched among marker genes for chondromyocytes, fibromyocytes, and SMC1, indicating a phenotypic shift toward these states. This transition appears to progress over time, with more overlapping genes at 8 weeks than at 2 weeks following YAP/TAZ deletion (Fig. 5C).

The greatest overlap was observed between the 8-week YT profile and the chondromyocyte gene signature, with 265 overlapping genes, suggesting a pronounced shift toward a chondrogenic phenotype. This gene set included key regulators of chondrogenic transdifferentiation such as *Sox9, Col2a1, Col11a1, Col9a2, Acan* (Aggrecan), and *Prg4*. Additional upregulated genes included *Col8a1* and *Anxa8*, associated with extracellular matrix remodeling, as well as *Spp1* and *Lcn2*, which are involved in inflammatory and osteogenic signaling pathways. Together, these findings support a dynamic, progressive reprogramming of VSMCs toward a chondromyocyte-like phenotype in response to sustained YAP/TAZ loss.

A key feature of atherosclerosis pathogenesis is the phenotypic switching of VSMCs, in which cells adopt fibroblast- or chondrocyte-like characteristics and contribute to extracellular matrix remodeling. Carramolino et al. [17] reported that following cholesterol-lowering therapy, both chondromyocyte and fibromyocyte populations within atherosclerotic plaques exhibited a reduction in inflammatory gene expression (*Lbp, Hp, Lcn2, Cxcl1, Nos2*), diminished extracellular matrix remodeling activity (*Col2a1, Col11a1, Spp1, Sox9*), and a partial re-expression of contractile smooth muscle markers (*Acta2, Myl9, Lmod1, Tagln*). Notably, many of these differentially expressed genes were part of the YAP/TAZ regulon and were regulated in the opposite direction in the 8-week YT (Fig. 5D). These findings suggest that YAP/TAZ-dependent transcriptional programs may be at least partially reversible upon lipid-lowering treatment, highlighting a potential link between cholesterol metabolism and VSMC phenotypic plasticity.

### 3.3 Shared gene signature in YAP/TAZ-deficient VSMCs and atherosclerosis in human

**Fig. 6:**
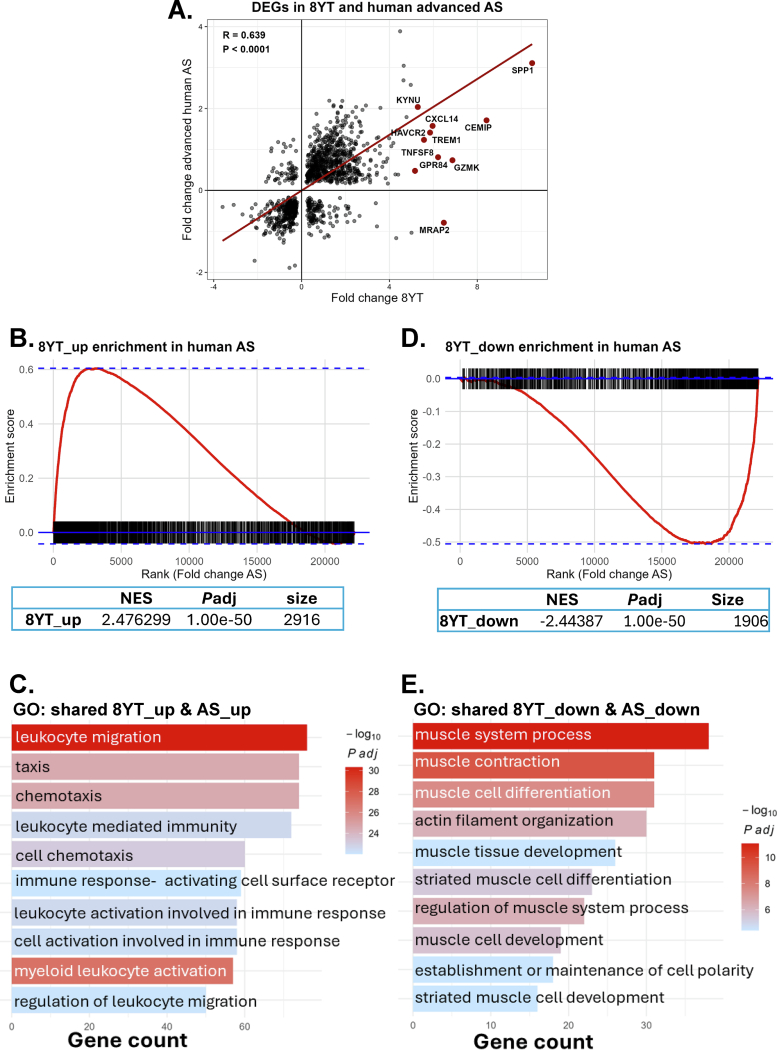
**Comparison of transcriptomic programs in 8-week VSMCs-specific YAP/TAZ knockout mice versus human advanced atherosclerotic plaques.** **(A)** Scatter plot showing the correlation between log₂fold changes of DEGs in 8YT (x-axis) and corresponding DEGs identified in human advanced atherosclerotic lesions (y-axis). Linear regression line and correlation coefficient are depicted. The most shared upregulated genes highlighted in red. **(B)** Gene set enrichment analysis (GSEA) of 8YT-upregulated genes within the ranked list of all genes from human AS (ranked by log₂FC). The enrichment score (red curve) peaks toward the left side of the x-axis (highly upregulated in AS). **(C)** Bar plot of GO biological processes enriched among genes that are upregulated both in 8YT and in human AS. Bars are ordered by gene count (x-axis), and the color scale reflects −log₁₀Padj. (D) GSEA of 8YT-downregulated genes against the human AS gene ranking. (E) Bar plot of GO biological processes enriched among genes downregulated both in 8YT and in human AS. AS, atherosclerosis; DEGs, differentially expressed genes; GSEA, gene set enrichment analysis; NES, normalized enrichment score; Padj, Benjamini–Hochberg adjusted P value; GO, Gene Ontology. (For interpretation of the references to color in this figure legend, the reader is referred to the web version of this article.)

To determine whether the transcriptional changes observed in YAP/TAZ-deficient VSMCs are relevant to human atherosclerosis, we reanalyzed a published dataset of gene expression profiles in human carotid artery plaques from early and advanced lesions (GSE28829) [28]. We first compared the 8-week YT expression profile to the human dataset and found a positive correlation between DEGs expressed in 8-week YT and those in advanced atherosclerotic plaques (Fig. 6A), suggesting conservation of gene regulation across species and disease models. Notably, genes such as *TREM2, CXCL16*, and *CCR1* were among the top contributors to these pathways, highlighting a convergence between YAP/TAZ loss and inflammatory activation observed in late-stage human plaques.

We performed GSEA using ranked gene expression changes from human advanced versus early atherosclerotic plaques and assessed the enrichment of genes upregulated or downregulated in the 8-week YT. Strikingly, genes upregulated in 8-week YT were significantly enriched in advanced plaques (NES = 2.48, Padj = 1.0 × 10^−50^), suggesting that YAP/TAZ loss activates a transcriptional program that parallels late-stage plaque remodeling in humans (Fig. 6B). GO enrichment analysis of the shared upregulated genes revealed strong enrichment for processes related to leukocyte migration, immune response, and chemotaxis, highlighting the pro-inflammatory and immunomodulatory nature of the YAP/TAZ-deficient state (Fig. 6C). Supporting this, Reactome pathway analysis identified significant enrichment of immune-related signaling pathways, including immune system, innate immune system, and cytokine signaling, involving over 300 overlapping genes (Table 5, Table S5). These results underscore the profound immune activation associated with the loss of YAP/TAZ.

**Table 5:** Reactome pathway analysis of overlapping upregulted genes between 8-week YT, and human atherosclerosis (Human_AS_1), showing the top 6 pathways.

Pathway identifier	Pathway name	Number of genes	FDR
R-HSA-168256	Immune System	310	2.68E-14
R-HSA-162582	Signal Transduction	204	2.69E-03
R-HSA-168249	Innate Immune System	153	2.68E-14
R-HSA-1280215	Cytokine Signaling in Immune system	147	2.68E-14
R-HSA-449147	Signaling by Interleukins	112	2.68E-14
R-HSA-1280218	Adaptive Immune System	97	1.74E-08

Conversely, genes downregulated in 8-week YT were significantly depleted in advanced plaques (NES = −2.44, Padj = 1.0 × 10^−50^), indicating that the contractile gene program is suppressed in both YAP/TAZ-deficient VSMCs and advanced human lesions (Fig. 6D). Functional annotation of these shared downregulated genes pointed to processes such as muscle contraction, actin cytoskeleton organization, and muscle cell differentiation (Fig. 6E). Consistently, Reactome pathway analysis highlighted significant downregulation of pathways like signaling by Rho GTPases and muscle contraction, reflecting impaired contractile machinery (Table 6, Table S6). This gene set included canonical VSMC markers such as *ACTA2*, *TPM1*, *LMOD1*, and *ROCK1*, reinforcing the loss of classical smooth muscle identity upon YAP/TAZ deletion.

**Table 6:** Reactome pathway analysis of overlapping downregulated genes between 8-week YT and human atherosclerosis (Human_AS_1), showing the top 6 pathways.

Pathway identifier	Pathway name	Number of genes	FDR
R-HSA-9716542	Signaling by Rho GTPases, Miro GTPases and RHOBTB3	36	1.07E-02
R-HSA-194315	Signaling by Rho GTPases	34	2.00E-02
R-HSA-397014	Muscle contraction	25	1.41E-06
R-HSA-5576891	Cardiac conduction	13	1.35E-02
R-HSA-445355	Smooth Muscle Contraction	11	1.92E-04
R-HSA-5578775	Ion homeostasis	11	2.28E-04

Together, these findings suggest that YAP/TAZ deletion in VSMCs induces a transcriptomic shift toward an inflammatory, less differentiated phenotype that closely mirrors transcriptional changes seen during human atheroprogression.

### 3.4 Identification of direct YAP/TAZ transcriptional targets

A central question remains: which of the dysregulated genes following YAP/TAZ deletion are direct transcriptional targets mediated through TEAD binding, and which represent secondary effects arising from phenotypic switching or inflammatory signaling? Distinguishing direct targets is crucial for elucidating the core molecular mechanisms governed by YAP/TAZ in VSMCs. To address this, we integrated publicly available ChIP-seq datasets for YAP and TEAD1–4 from both human and mouse sources with promoter motif enrichment analyses for TEAD-binding elements. We then intersected this curated regulatory dataset with genes that were consistently downregulated at both 2 and 8 weeks following VSMC-specific YAP/TAZ deletion. This integrative strategy identified a subset of 19 genes likely to be directly regulated by the YAP/TAZ–TEAD complex (Table 7).

**Table 7:** 19 putative YAP/TAZ–TEAD target genes defined by our cross‐species ChIP‐seq and promoter‐ Tead motif scanning. Each row lists one gene, with the highest promoter‐associated ChIP‐seq peak score observed in human and mouse datasets, color‐coded using a five‐tier scheme: blue (< 200), cyan (200–499), green (500–749), yellow (750–999), and red (≥ 1000). “Presence of TEAD motif” indicates whether at least one TEAD‐binding site (from JASPAR2022 TEAD1–4 PWMs) was found within ±2 kb of the transcription start site in either species. All 19 genes are also significantly downregulated in VSMCs-specific YAP/TAZ knockout mice at both 2 weeks and 8 weeks (2YT and 8YT).

YAP/ TAZ-TEAD Predicted targets	Peak score (Human)	Peak score (Mouse)	Presence of TEAD_motif
*Fgfr2*	green	red	TRUE
*Ptpn21*	red	cyan	TRUE
*Plxna4*	red	cyan	TRUE
*Fxyd7*	cyan	red	TRUE
*Gpsm1*	red	yellow	TRUE
*Ntrk3*	green	green	TRUE
*Npas2*	green	green	TRUE
*Cirbp*	yellow	cyan	TRUE
*Plekhg2*	red	cyan	TRUE
*Kif1c*	green	green	TRUE
*Srf*	green	green	TRUE
*Gpn1*	red	green	TRUE
*Limch1*	red	green	TRUE
*Thap3*	green	red	TRUE
*Fas*	cyan	green	TRUE
*Map2k6*	cyan	cyan	TRUE
*Pcif1*	red	yellow	TRUE
*Aif1l*	green	cyan	TRUE
*Nexn*	red	cyan	TRUE

**Fig. 7:**
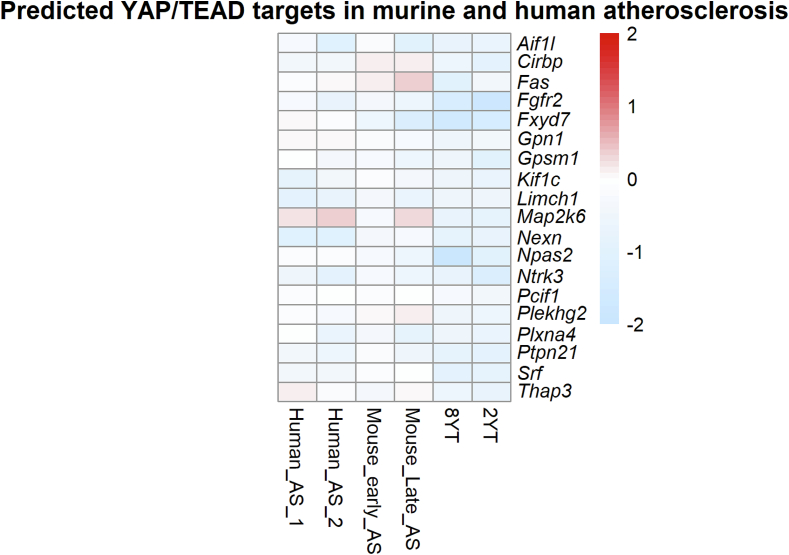
**YAP/TAZ–TEAD predicted targets in atherosclerosis.** Heatmap showing log₂fold change (log₂FC) for 19 predicted YAP/TAZ–TEAD targets across murine and human atherosclerosis datasets. Murine datasets: (GSE240233) VSMC-specific YAP/TAZ knockout RNA-seq at 2 weeks (2YT) and 8 weeks (8YT) after tamoxifen induction; (GSE205929) Mouse_early_AS (1-month) and Mouse_late_AS (3-month) aortic lesions from pro-atherogenic mice on high-fat diet. Human datasets: advanced carotid plaque vs early lesion (GSE28829) Human_AS_1, and Human_AS_2 [19]. 8YT: aorta from YAP/TAZ-deficient mice 8 weeks after tamoxifen induction. 2YT: aorta from YAP/TAZ-deficient mice 2 weeks after tamoxifen induction. AS, atherosclerosis; VSMC, vascular smooth muscle cell; YT, YAP/TAZ VSMC-specific knockout; log₂FC, log₂fold change; Padj, Benjamini–Hochberg adjusted P value.

To evaluate how these putative direct targets are modulated in atherosclerosis, we analyzed the expression profiles of the 19 YAP/TAZ–TEAD target genes across both murine and human atherosclerosis datasets (Fig. 7). Serum response factor (*Srf*), a master regulator of smooth muscle cell differentiation, was significantly downregulated, particularly in human atherosclerosis, along with its downstream effector Nexilin (*Nexn*), an actin filament–binding protein critical for cytoskeletal integrity [29]. Several additional targets exhibited consistent downregulation across atherosclerosis datasets, including genes involved in cytoskeleton organization and actin dynamics (*Limch1, Kif1c, Aif1l, Ptpn21*), signal transduction as kinases or receptors (*Plxna4, Fgfr2, Gpsm1, Ntrk3*), transcriptional regulation (*Npas2, Gpn1*), and ion transport modulation (*Fxyd7*, a regulator of Na^+^/K^+^-ATPase activity).

Collectively, this integrated transcriptomic and regulatory analysis reveals specific molecular candidates that link disruption of YAP/TAZ–TEAD signaling in VSMCs to the pathogenesis of atherosclerosis. These findings underscore the dual role of YAP/TAZ in suppressing pro-inflammatory programs while sustaining the contractile phenotype, providing insight into how YAP/TAZ loss may promote vascular inflammation, matrix remodeling, and SMC dedifferentiation, all key features of atherosclerosis.

## 4 Discussion

This study demonstrates that VSMC-specific deletion of YAP and TAZ recapitulates key features of transcriptional reprogramming observed during atherosclerosis progression, notably inflammatory activation and phenotypic switching. These findings support the emerging view of VSMCs as dynamic contributors to atherosclerosis and underscore the central role of YAP/TAZ in maintaining vascular homeostasis [3].

Our transcriptomic analyses revealed substantial overlap between gene expression profiles in YAP/TAZ-deficient VSMCs and murine models of both early and advanced atherosclerosis. While the percentage of overlapping genes was higher with early-stage atherosclerosis, the absolute number of overlapping genes was markedly greater with advanced disease, particularly following prolonged YAP/TAZ deletion (8-week YT). This pattern reflects a progressive transcriptional reprogramming induced by YAP/TAZ loss that increasingly mirrors the molecular landscape of atherosclerosis development.

GO enrichment and Reactome pathway analyses highlighted upregulation of immune-related pathways, including leukocyte migration and extracellular matrix remodeling. These findings are consistent with increased immune cell infiltration and extracellular matrix degradation in advanced plaques [30]. Shared upregulated genes included established inflammatory mediators such as osteopontin (*Spp1*), serum amyloid A3 (*Saa3*), lipocalin 2 (*Lcn2*), and *Cxcl4*, known to contribute to vascular inflammation and immune cell recruitment [[31], [32], [33], [34]]. Advances in lineage tracing and scRNA-seq have enabled the detailed characterization of VSMC-derived cell states in atherosclerosis [3]. In this context, the transcriptional profile of YAP/TAZ-deficient VSMCs closely resembled that of chondromyocyte-like cells, consistent with earlier observation of *Sox9* upregulation and cartilage-like matrix accumulation in the media of YAP/TAZ-deficient arteries [10]. Previous work has linked VSMC transdifferentiation toward osteo-chondrogenic fate with vascular calcification in advanced plaques [35]. Carramolino et al. further demonstrated that reducing ApoB via cholesterol-lowering therapy diminished the chondromyocyte-like population and restored contractile gene expression in atherosclerotic vessels [17]. These changes were opposite to those observed in YAP/TAZ-deficient VSMCs, reinforcing the protective role of YAP/TAZ in preserving contractile identity and limiting disease-associated remodeling.

Our analysis of human datasets further underscores the translational relevance of these findings. Gene expression profiles from advanced human carotid plaques significantly correlated with those from YAP/TAZ-deficient mouse models, particularly with respect to inflammation and extracellular matrix remodeling. Key genes, including *SPP1, CEMIP,* and *CXCL14* were consistently upregulated in both mouse and human datasets.

To identify genes directly regulated by YAP/TAZ, we integrated ChIP-seq data with TEAD-binding motif analysis, uncovering 19 possible YAP/TAZ–TEAD target genes that were consistently downregulated following YAP/TAZ deletion in VSMCs. Among these, SRF, a key regulator of VSMC phenotype, emerged as a notable finding. SRF governs the transcription of numerous cytoskeletal and contractile genes and is essential for maintaining the differentiated, contractile state of VSMCs [36]. Its transcriptional activity depends critically on its co-activator myocardin, which enhances SRF-mediated expression of smooth muscle–specific genes [37]. Disruption of the SRF–myocardin axis has been mechanistically linked to VSMC phenotypic switching that contributes to fibrous cap thinning, extracellular matrix degradation, and heightened plaque vulnerability in atherosclerosis [38]. Moreover, SRF-myocardin axis has been shown to be antagonized by the over-expression of the coronary artery disease-risk gene *Tcf21* in vivo [39]. One SRF transcriptional target is nexilin, an F-actin–binding protein, which was also downregulated in atherosclerosis datasets. Nexilin contributes to the structural integrity of the contractile VSMCs by reinforcing the cortical actin cytoskeleton and is recognized as a smooth muscle marker [29].

In addition to SRF and nexilin, many of the other YAP/TAZ–TEAD targets were likewise downregulated in atherosclerosis datasets, reinforcing the notion that YAP/TAZ deficiency recapitulates disease-associated transcriptional reprogramming. These included genes involved in cytoskeletal scaffolding and stability (*Limch1, Kif1c, Aif1l, Ptpn21*), receptor-mediated signaling (*Plxna4, Fgfr2, Gpsm1, Ntrk3*), and transcriptional control (*Npas2, Gpn1*). Of note, *Plxna4*, a semaphorin receptor involved in cytoskeletal remodeling and vascular permeability, has been shown to be reduced in coronary artery disease, with its suppression linked to increased vascular inflammation and endothelial barrier dysfunction [40]. Additionally, Salido et al. reported that *Limch1* is transcriptionally suppressed in human plaques and carriers of the 9p21.3 coronary artery disease risk allele, linking its loss to VSMC osteochondrogenic reprogramming and enhanced susceptibility to atherosclerosis [41].

While our findings support a protective role for YAP/TAZ signaling in VSMCs, their function appears to be context dependent. For instance, Xu et al. demonstrated that in endothelial cells, atheroprotective laminar flow suppresses YAP activation, whereas disturbed flow, prevalent at branch points, induces YAP nuclear translocation and activation [42]. Thus, the vascular impact of YAP/TAZ is likely to vary across different cell types and hemodynamic environments.

## 5 Conclusion

In conclusion, our study adds to growing evidence that YAP/TAZ signaling supports the contractile, anti-inflammatory identity of VSMCs and opposes transcriptional programs that drive phenotypic switching and plaque progression. The transcriptional convergence observed between YAP/TAZ-deficient VSMCs and atherosclerotic lesions suggests that loss of YAP/TAZ activity contributes to disease evolution. However, our findings are primarily based on transcriptomic analyses and correlative associations. Functional validation of key YAP/TAZ–TEAD targets in vivo, including human specimens will be necessary to establish direct causal roles. Future studies should also investigate whether modulating YAP/TAZ activity in VSMCs can reverse maladaptive phenotypes or stabilize pre-existing atherosclerotic lesions. Together, these findings highlight the therapeutic potential of targeting YAP/TAZ signaling to maintain vascular integrity and mitigate atherogenesis.

The following is the supplementary data related to this article.

Supplementary Table 1 Supplementary Table 1

## CRediT authorship contribution statement

**Fatima Daoud:** Writing – review & editing, Writing – original draft, Visualization, Methodology, Formal analysis, Data curation, Conceptualization. **Johan Holmberg:** Writing – review & editing, Conceptualization. **Hanna Winter:** Writing – review & editing, Resources. **Nadja Sachs:** Writing – review & editing, Resources. **Lars Maegdefessel:** Writing – review & editing, Resources. **Sebastian Albinsson:** Writing – review & editing, Visualization, Supervision, Software, Resources, Project administration, Methodology, Funding acquisition, Conceptualization.

## Funding

This work was supported by grants from The 10.13039/501100009708Novo Nordisk Foundation (NNF22OC0078361), The 10.13039/501100003793Swedish Heart and Lung Foundation (20220527), The 10.13039/501100006189Albert Påhlsson Foundation, and The Thure Carlsson Memorial Foundation, Gyllenstiernska Krapperups Foundation.

## Declaration of competing interest

We have no competing interests to declare.
